# Acupuncture Activates ERK-CREB Pathway in Rats Exposed to Chronic Unpredictable Mild Stress

**DOI:** 10.1155/2013/469765

**Published:** 2013-06-17

**Authors:** Jun Lu, Jia Liang, Jun-Ren Wang, Li Hu, Ya Tu, Jian-You Guo

**Affiliations:** ^1^School of Acupuncture-Moxibustion and Tui Na, Beijing University of Chinese Medicine, Beijing 100029, China; ^2^Graduate School of China Academy of Chinese Medical Sciences, Beijing 100700, China; ^3^Beijing Heping Hospital of Chaoyang District, Beijing 100103, China; ^4^Key Laboratory of Mental Health, Institute of Psychology, Chinese Academy of Sciences, Beijing 100101, China

## Abstract

Extracellular signal-regulated kinase (ERK)-cAMP response element binding protein (CREB) signal pathway has been implicated in the pathogenesis of depression. There is growing evidence that acupuncture in traditional Chinese medicine has antidepressant-like effect. However, the effect of acupuncture on ERK-CREB pathway remains unknown. In our study, the antidepressant-like effect of acupuncture treatment was measured by sucrose intake test and open field test in rats exposed to chronic unpredictable mild stress (CUMS) for 4 weeks. The protein levels of ERK1/2, CREB, phosphorylated ERK1/2 (p-ERK1/2), and phosphorylated CREB (p-CREB) in the hippocampus (HP) and prefrontal cortex (PFC) were examined by Western blot analysis. Our results showed that CUMS rats exhibited the reduction in behavioral activities, whereas acupuncture stimulation at acupoints Baihui (Du20) and Neiguan (PC6) reversed the behavioral deficit. In addition, exposure to CUMS resulted in the decrease of p-ERK1/2 and p-CREB in the HP and PFC. Acupuncture increased the ratio of p-ERK1/2 to ERK1/2 and the ratio of p-CREB to CREB in the HP and PFC. Our study suggested that one potential way, by which acupuncture had antidepressant-like effect, might be mediated by activating the ERK-CREB pathway in the brain.

## 1. Introduction

Depression is the leading cause of disability and the 4th leading contributor to the global burden of disease in the world, with a lifetime prevalence of up to 17% [1]. Although antidepressant medications represent the mainstay treatment for depression, almost one-fourth of patients fail to respond to the treatment [2]. Attempts have been made to seek alternative medicine for treatment options [3]. In Chinese medicine, acupuncture has been widely used to treat depression [4, 5]. However, the mechanism underlying the acupuncture treatment in depression remains unclear.

The extracellular signal-regulated kinase (ERK)-cAMP response element binding protein (CREB) signal pathway is implicated in learning, memory, and neuroplasticity [6] and plays an important role in regulating many brain functions, including cell growth, differentiation, apoptosis, and cellular responses to stress [7]. It has been reported that ERK-CREB signal pathway involves in stress response and depression [8]. For example, previous reports have indicated that chronic stress reduced phosphorylated ERK (p-ERK) and phosphorylated CREB (p-CREB) expression in rat brain [9, 10]. However, little is known about whether acupuncture could affect ERK-CREB signal pathway in stress and depression.

Therefore, the primary goal of the present study was to evaluate the effect of acupuncture on ERK-CREB signal pathway in a chronic unpredictable mild stress (CUMS) rat model of depression. The model is a widely accepted rodent model of depression, which employs various stressors to mimic some symptoms of depression in humans [11]. We investigated the effects of acupuncture on the behavioral activities and detected protein levels of ERK1/2, CREB, p-ERK1/2, and p-CREB in the hippocampus (HP) and prefrontal cortex (PFC) by using Western blot analysis. The HP and PFC are thought to be involved in reward and affective circuitry and play a major role in mood regulation and the pathophysiology of depression [12, 13].

## 2. Materials and Methods

### 2.1. Animals

Male Sprague-Dawley rats (180~200 g) were obtained from Beijing Vital River Laboratories. Rats were kept in an air-conditioned room with a 12 h light/dark cycle with free access to food and water except when animals were subjected to deprivation stressors as described in stress. The experiment procedures were approved by the Animal Care and Use Committee at Beijing University of Chinese Medicine. 

### 2.2. Groups and Treatment

The rats were randomly divided into five groups (ten rats in each group): the Normal group was given no stress except general handling for 4 weeks; the Normal + Acu group was treated with the same as the Normal group but with acupuncture stimulation; the CUMS group was exposed to CUMS for 4 weeks; the CUMS + Acu group received acupuncture treatment once every other day during the 4-week stress period; the CUMS + Paroxetine (Par) group, used as a positive comparator for an antidepressant effect, was given once daily oral gavage (p.o.) administration of Paroxetine (10 mg/kg, GSK Co., Ltd, China) during the 4-week stress period. Paroxetine was diluted in distilled water and orally given one hour before the stress exposure. The dosage of 10 mg/kg for paroxetine has been reported to show antidepressant action in previous work [14, 15].

### 2.3. Chronic Unpredictable Mild Stress Procedure

Rats in stressed groups were exposed to CUMS after 1 week of acclimatization period under the housing conditions. The CUMS model was modified from the methods previously described [11, 16]. Six different stressors were used to induce a depressive state, including food deprivation (24 h), water deprivation (24 h), cold water swimming (4°C, 5 min), cage shaken on a rocking bed (30 min; ZD-9556, manufactured in Taicang Scientific Instruments Limited, China), behavior restraint on a restraining device (3 h), and tail clip (2 min). These stressors lasted for 4 weeks, and a different stressor was administered on each day. The tail clip was used twice in a week. For example, the following stressors were arranged in one week: Day 1 swim, Day 2 tail clip, Day 3 food deprivation, Day 4 shaken on a rocking bed, Day 5 restraint, Day 6 tail clip, and Day 7 water deprivation. The stressors were arranged semirandomly to make the last stressor be water deprivation. The stress sequence was changed every week in order to make the stress procedure unpredictable. 

### 2.4. Acupuncture Treatment

During acupuncture administration, the rats were maintained within a cloth bag with one forelimb taken out by an assistant, similar to what we described previously [17]. Sterilized disposable stainless steel needles of 0.3 mm diameter were inserted as deep as 2-3 mm at Baihui (GV20) and Neiguan (PC6). GV20 is located above the apex auriculate, on the midline of the head. PC6 is located between the tendons of m. palmaris longus and m. flexor carpi radialis, proximal to the transverse crease of the wrist. The acupuncture treatment was manually delivered by twisting the acupuncture needles at a frequency of twice per second for 1 minute, and then the needles were retained for 10 minutes. The rats received acupuncture treatment once every other day during the 4-week period.

### 2.5. Behavior Tests

The sucrose intake test was a modified version from the literature [18, 19]. The 5% sucrose solution was previously described [19]. Before the test, the rats were habituated to consume 5% sucrose solution for 24 h without any water available. On the last stressed day, the rats were deprived of water for 24 h. Then the rats were given a 1 h window sucrose test (between 14:00 and 15:00 h). The sucrose consumption was measured by reweighing preweighed bottles of sucrose solution. 

At the end of the experiment, the open field test was performed. The apparatus consisted of a square arena 80 × 80 cm with 40 cm high wall. It was divided into 25 × 25 equal squares which had been drawn in the floor of the arena. A single rat was gently placed in the center of the floor in order to explore the arena for 5 min. The crossing numbers (defined as at least three paws in a square) and the rearing numbers (defined as the rat standing upright on its hind legs) were counted manually by two observers who were blind to the experiment. The body weight was measured on day 1 and day 28 of the experiment. 

### 2.6. Western Blot Analysis

After the behavioral tests were completed, six rats in each group were sacrificed by decapitation for Western blot analysis. Then the HP and PFC were dissected and put into chilled tubes treated with an enzyme inhibitor. Brain tissue was homogenized and Western blot analysis carried out as previously reported [20], using primary antibodies for rabbit ERK1/2, rabbit p-ERK1/2, rabbit CREB, rabbit p-CREB, and *β*-actin at 1 : 1000 dilution (Santa Cruz Biotech Inc., CA, USA). A secondary antibody conjugated with horseradish peroxidase was used. Immunoreactivity was visualized by ECL reagent. ERK1/2, p-ERK1/2, CREB, and p-CREB protein expression were quantified by densitometry using the Scion Image Beta 4.02 software and are shown as density relative to *β*-actin.

### 2.7. Statistical Analysis

Data were presented as means ± S.E.M. Differences among groups were examined using one-way ANOVA, followed by Newman-Keuls test. *P* < 0.05 was the accepted level of significance.

## 3. Results

### 3.1. Effects of Acupuncture Treatment on the Body Weight

**Figure 1:**
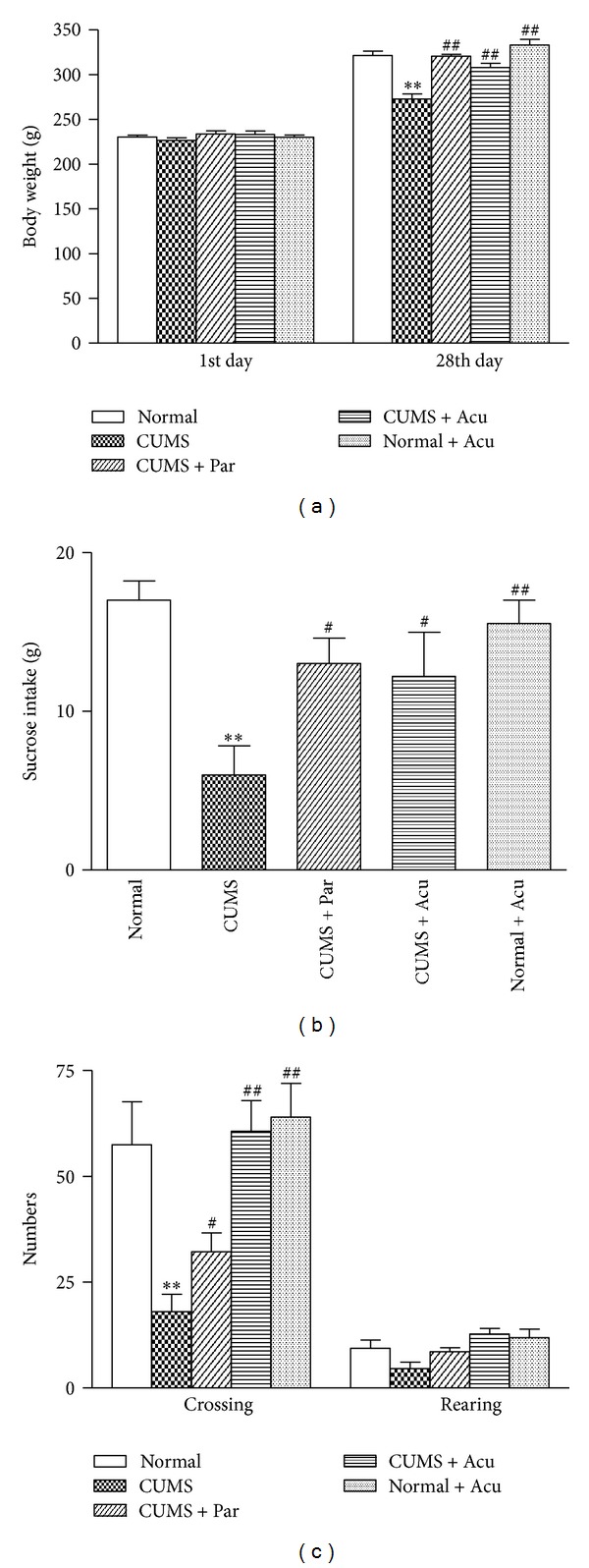
The effect of acupuncture on body weight, sucrose intake test, and locomotor activity in open field test in the following groups (*n* = 10 per group): Normal, CUMS, CUMS + Par, CUMS + Acu, and Normal + Acu. (a) Body weight. (b) Sucrose intake test. (c) Open field test. **P < 0.01 as compared with the Normal group, ^#^ *P* < 0.05, ^##^ *P* < 0.01 as compared with the CUMS group.

As shown in Figure 1(a). At the beginning of the experiment, there was no significant difference among groups in the body weight [*F*_(4,45)_ = 0.90, *P* > 0.05]. After 28-day stress procedure, significant difference was observed among groups [*F*_(4,45)_ = 21.35, *P* < 0.01]. The CUMS rats showed a significant decrease of the body weight compared to normal rat (*P* < 0.01). Acupuncture treatment and paroxetine had significant effect on the decrease in the body weight (*P* < 0.01, either). There was no significant difference in the body weight between the normal rats and Normal + Acu rats (*P* > 0.05), suggesting that acupuncture had no effect on the body weight in normal rats.

### 3.2. Effects of Acupuncture Treatment on the Sucrose Intake Test

 The sucrose intake test is used to predict sensitivity to rewards [21]. As seen in Figure 1(b), the sucrose solution intake significantly differed among groups after stress procedure [*F*_(4,45)_ = 5.23, *P* < 0.01]. The sucrose intake was significantly reduced in CUMS group compared to Normal group (*P* < 0.01), suggesting the anhedonia was induced by CUMS. Paroxetine and acupuncture treatment increased the decrease in sucrose intake caused by CUMS (*P* < 0.05, either), which is indicative of an increasing rewarding effect of sucrose solution after treatment. There was no significant difference in the sucrose solution intake between the normal rats and Normal + Acu rats (*P* > 0.05), suggesting that acupuncture treatment had no effect on the sucrose solution intake in the naive rats.

### 3.3. Effects of Acupuncture Treatment on the Open Field Test

 The open field test was used to study the exploratory and locomotor activity [22, 23]. As seen in Figure 1(c), in the open field test, there was significant difference among groups in the number of crossings after stress procedure [*F*_(4,45)_ = 8.03, *P* < 0.01]. In comparison to normal rats, CUMS rats showed a significant reduction of crossings (*P* < 0.01) but not rearings (*P* > 0.05). Acupuncture treatment and paroxetine improved the locomotor activity decreased by CUMS (*P* < 0.01, 0.05, resp.). There was no significant difference in the rearings between the normal rats and CUMS rats (*P* > 0.05). There was no significant difference in the crossings between the normal rats and Normal + Acu rats (*P* > 0.05), suggesting that acupuncture treatment had no effect on the locomotor activity in the naive rats.

### 3.4. Effects of Acupuncture Treatment on the ERK1/2 and p-ERK1/2 Levels in the HP and PFC

**Figure 2:**
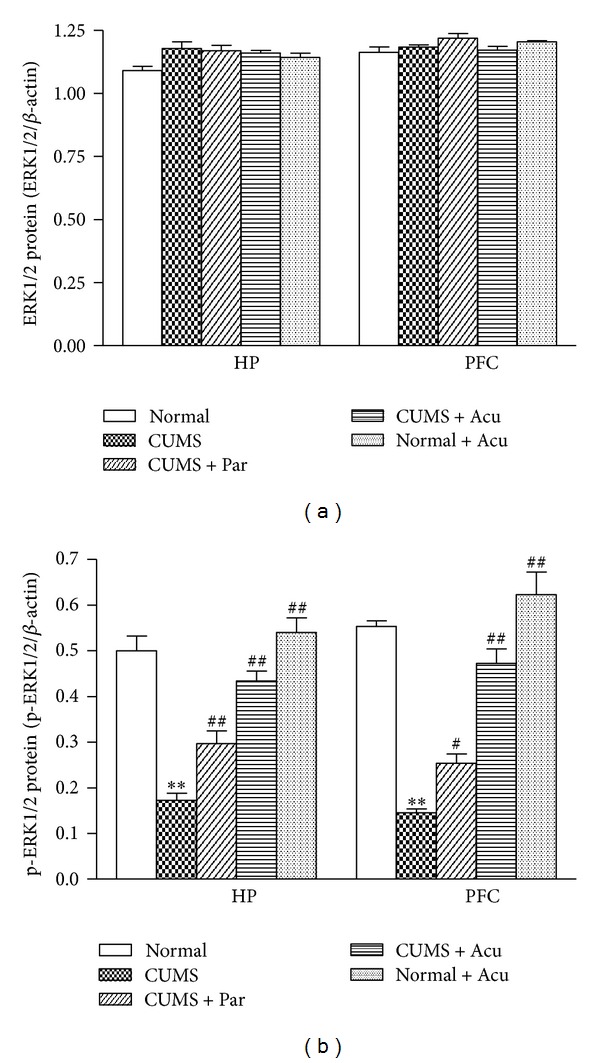
The effect of acupuncture on ERK1/2 and p-ERK1/2 protein expression in hippocampus (HP) and prefrontal cortex (PFC) in the following groups (*n* = 6 per group): Normal, CUMS, CUMS + Par, CUMS + Acu, and Normal + Acu. (a) ERK1/2; (b) p-ERK1/2. **P < 0.01 as compared with the Normal group, ^#^ *P* < 0.05, ^##^ *P* < 0.01 as compared with the CUMS group.

Western blot analysis revealed that there was no significant difference in the ERK1/2 protein level among groups in the HP [*F*_(4,25)_ = 3.44, *P* > 0.05] and the PFC [*F*_(4,25)_ = 2.17, *P* > 0.05], as seen in Figures 2(a) and 4. However, p-ERK1/2 level significantly differed among groups in the HP [*F*_(4,25)_ = 32.02, *P* < 0.01] and PFC [*F*(4, 25) = 49.71, *P* < 0.01]. Chronic stress significantly decreased p-ERK1/2 in the HP (*P* < 0.01) and PFC (*P* < 0.01) compared to Normal group. Acupuncture treatment and paroxetine significantly increased p-ERK1/2 level in HP (*P* < 0.01, either) and PFC (*P* < 0.01, either). There was no significant difference in p-ERK1/2 between the Normal and Normal + Acu groups, suggesting that acupuncture had no effect on ERK1/2 activity in the naive rats, as seen in Figures 2(b) and 4.

### 3.5. Effects of Acupuncture Treatment on the CREB and p-CREB Levels in the HP and PFC

**Figure 3:**
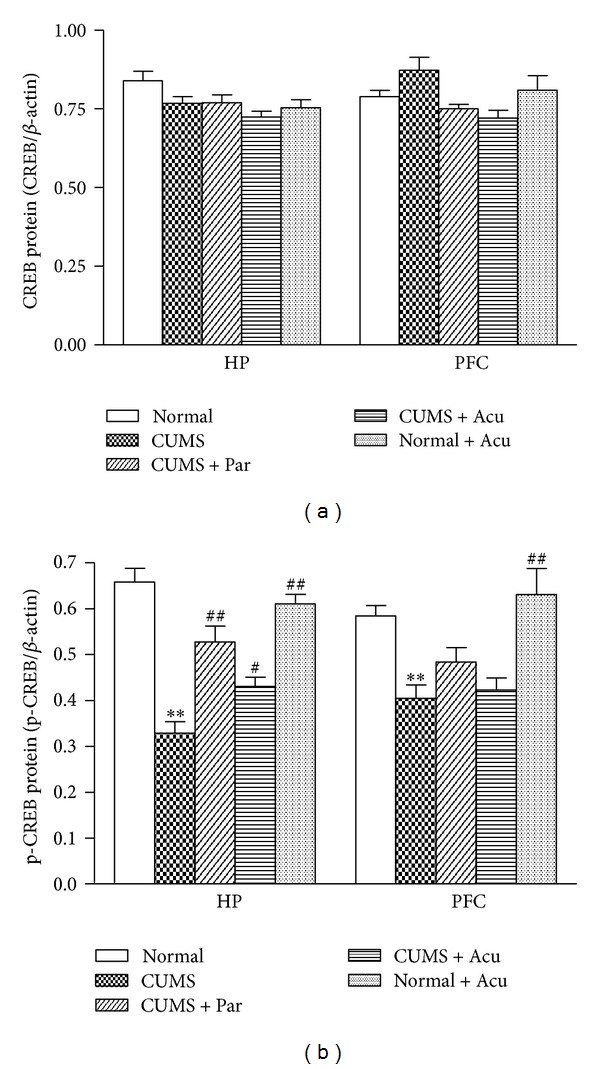
The effect of acupuncture on CREB and p-CREB protein expression in hippocampus (HP) and prefrontal cortex (PFC) in the following groups (*n* = 6 per group): Normal, CUMS, CUMS + Par, CUMS + Acu, and Normal + Acu. (a) CREB; (b) p-CREB. **P < 0.01 as compared with the Normal group, ^#^ *P* < 0.05, ^##^ *P* < 0.01 as compared with the CUMS group.

**Figure 4:**
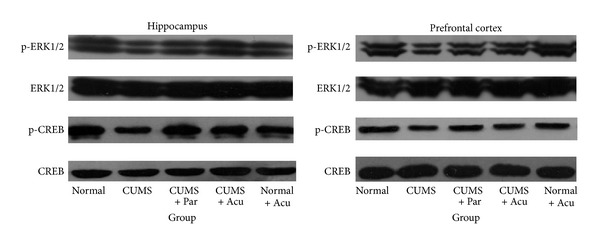
Representative Western blots showing levels of p-ERK1/2, ERK1/2, p-CREB, and CREB in the hippocampus and prefrontal cortex of the following groups (*n* = 6 per group): Normal, CUMS, CUMS + Par, CUMS + Acu, and Normal + Acu.

The CREB protein level did not significantly differ among groups in the HP [*F*_(4,25)_ = 3.08, *P* > 0.05] and the PFC [*F*_(4,25)_ = 3.39, *P* > 0.05], as seen in Figures 3(a) and 4. However, p-CREB protein level significantly differed among groups in the HP [*F*_(4,25)_ = 24.98, *P* < 0.01] and PFC [*F*_(4,25)_ = 7.83, *P* < 0.05]. Chronic stress significantly decreased p-CREB in the HP and PFC (*P* < 0.01, either) compared to Normal group. Acupuncture treatment and paroxetine significantly increased the p-CREB level in HP (*P* < 0.01, 0.05, resp.) but not in PFC (*P* > 0.05), as seen in Figures 3(b) and 4.

## 4. Discussion

The major finding of the present study is that chronic stress exposure caused deficits in ERK and CREB activation in the HP and PFC, which could be reversed by acupuncture treatment. We also found that the behavior deficits induced by chronic stress were restored by acupuncture. Our results suggested that acupuncture could activate ERK-CREB pathway in the rats exposed to CUMS, which might mediate the antidepressant-like effect of acupuncture.

The ERK-CREB signal pathway has been studied for its role in stress and depression [8, 24]. For example, preclinical studies have suggested that depression was associated with aberrant ERK signal pathway [25]. Chronic stress exposure caused the reduction in p-ERK and p-CREB in the HP of rat [9, 10]. On the other hand, controversial studies exist. For example, it has been reported that 14-day stress induced an increase in p-ERK1/2 and p-CREB in the HP [26]. The possible explanation for the discrepancy may lie in the difference in stress category, duration, and other experimental procedures. 

In consistency with most of these previous findings, we found that CUMS decreased p-ERK1/2 and p-CREB levels in the HP and PFC, without affecting ERK1/2 and CREB. It is well accepted that one of the most important properties of ERK1/2 activation is that it must be phosphorylated to exhibit full enzymatic activity. After activation, ERK induces nuclear translocation and phosphorylation of target transcription factors. CREB has been known to be a downstream of ERK and a critical transcription factor in regulating diverse processes such as neurodevelopment, neuronal plasticity, and survival [27–29]. ERK activation is necessary to induce the phosphorylation of the CREB and modulation of its transcriptional activity [30]. Therefore, it is suggested that chronic stress exposure caused deficits in ERK and CREB activation and inhibited ERK-CREB pathway in the brain, which could be implicated in depression.

Phosphorylation of ERK1/2 and CREB has been thought to be an intracellular signal mechanism mediating antidepressant effect [31, 32]. It has been found that antidepressants increased p-ERK and p-CREB in rats exposed to chronic stress [33]. In accordance with the previous results, our findings showed that acupuncture treatment increased p-ERK1/2 in the HP and PFC of CUMS rats and increased p-CREB in the HP. Our data showed that the ratio of p-CREB to CREB in the PFC and HP was significantly higher in CUMS + Acu group than that of CUMS rats (PFC: 0.59 ± 0.04 versus 0.46 ± 0.02, *P* < 0.05; HP: 0.60 ± 0.03 versus 0.43 ± 0.03, *P* < 0.05). Similarly, we found that acupuncture significantly increased the ratio of p-ERK to ERK in the PFC and HP when compared to CUMS rats (PFC: 0.40 ± 0.16 versus 0.12 ± 0.04, *P* < 0.01; HP: 0.37 ± 0.15 versus 0.14 ± 0.06, *P* < 0.01). Our results suggested that the upregulation of CREB activation is accompanied by the increase of ERK activation, which may suggest a role of the ERK-CREB pathway in the chronic effect of acupuncture.

We previously found that acupuncture increased the brain-derived neurotrophic factor (BDNF) protein level in the HP and PFC of rats exposed to chronic stress [34]. Phosphorylation of CREB is implicated in synaptic plasticity and regulates the transcription of the downstream genes encoding proteins, such as BDNF, c-fos, and many neuropeptides [35]. It is reasonable to speculate that there is a potential for acupuncture to influence BDNF expression and neuronal function by activating ERK-CREB pathway. 

In the present study, we found that acupuncture treatment slightly increased the protein levels of p-ERK1/2 and p-CREB in normal rats. Therefore, the traditional medicine acupuncture treatment seemed to induce a stimulation effect on the experimental animals, and traditional medicine might be beneficial in normal rat. Consistent with this finding, our previous study also showed that traditional medicine moxibustion might mildly affect the serum cytokines levels in control rat [17].

Paroxetine, a kind of selective serotonin reuptake inhibitors, is a clinically effective antidepressant drug. In accordance with previous finding [15], our results showed that paroxetine alleviated anhedonia induced by chronic stress. In addition, we found that paroxetine increased the levels of p-ERK1/2 and p-CREB in the hippocampus and PFC, which could partially mediate the antidepressant effect of paroxetine.

In the present study, the acupuncture was applied under a lightly restrained condition. Our previous work (data not published) showed that there was no significant difference in serum adrenocorticotropic hormone (ACTH) and corticosterone (Cort) level between the normal rats and Normal + Acu rats. It is suggested that the acupuncture administration will not induce stress response. 

## 5. Conclusions 

In conclusion, we found that chronic stress exposure decreased p-ERK1/2 and p-CREB in the rat brain and induced deficits in ERK1/2 and CREB activation. In addition, acupuncture treatment could activate ERK-CREB pathway and alleviate depressive-like behavior. Our results suggested that the antidepressant-like effect of acupuncture might be mediated by activating the ERK-CREB pathway in the brain.
